# Parliament and the Profession

**Published:** 1916-07-15

**Authors:** 


### PARLIAMENT AND THE PROFESSION.

### Certifying Surgeons and their Reports.

From the medical point of view by far the most interesting discussion in the House last week was that arising out of the clause in the Police Bill by which it was proposed to save the expense entailed by requiring a special report from factory certifying surgeons in the case of certain classes of accident. The Home Secretary, in moving the second reading of the Bill, said that the factory inspectors held an investigation in such cases, and the reports from certifying surgeons did not assist the inspectors at arriving at a conclusion as to how similar accidents might be prevented. The abolition of these reports would not increase the perils of workers in factories and workshops, but it would save £12,500 a year in fees to surgeons and a deal of unnecessary work upon inspectors.

In the course of his speech, Mr. Samuel asserted that the only opposition to this clause had come from the doctors themselves, but he soon discovered his mistake, for after he had resumed his seat one member after another opposed a proposal to abandon a system that had existed for over seventy years, Labour members being especially emphatic in their opposition. Mr. Samuel in his speech, in referring to the opposition of the doctors, observed that they were a strongly organised profession, and, proceeding, said: "Whenever there is any proposal which touches their interest they are very ready to approach members of the House of Commons. It is from them that circulars have been received which hon. members have had, and it is at their instigation, I venture to think, the opposition that has so far evinced itself proceeds. I do not know how at the present time, when there is so great a dearth of doctors, when we need medical men for the services of the war, we can defend requiring a number of medical men all over the country to spend time and energy in sending in 50,000 unnecessary reports. And if it is true that these gentlemen will no longer receive the fees to which they have been accustomed at the present time, when there is so great a demand for medical men, when the medical profession of this country is understaffed, this is the moment when the change can be made with the least hardship to the men who have hitherto performed this work." The latter part of this statement seemed to convey the impression that the proposal was only an emergency one, and Sir P. Magnus promptly asked, "Does this Bill provide for the period of the war only?" To which Mr. Samuel replied, "No; this proposal does not stop with the period of the war." The opinion of the Labour Party was expressed by various members; practically with one voice they opposed the Government proposals.

Some notion of the force of their arguments can be gained from the fact that when the Committee stage was reached the Home Secretary moved an amendment providing that certifying surgeons should continue to investigate and report upon cases of injury caused by exposure to gas, fumes, or other noxious substances, or due to any other special cause specified in instructions of the Secretary of State as requiring medical investigation. Sir P. Magnus moved an amendment to the amendment to enable certifying surgeons to make any report which they considered necessary. The clause was opposed by various Labour and other members, and a strong appeal was made to Mr. Samuel to withdraw it during the period of the war, but he said he was convinced the reports were useless and a sheer waste of money, and he could not reconcile it to his duty to give way. The amendment moved by Sir P. Magnus was negatived and the Government amendment was agreed to.

### Nervous Breakdown among Sailors.

Commander Bellairs asked the First Lord of the Admiralty what measures were being taken to deal with such cases of acute mental and nervous breakdown in the Royal Navy and Forces under the Admiralty as were unsuitable for treatment in general hospitals other than at Yarmouth Asylum; and whether, in view of the fact that the Director of the Medical Service had recently authorised an officer to give a full and detailed account of the methods of treatment for all ranks of the Army suffering from mental and nervous disorders, similar action would be taken in the Royal Navy so as to include in the report the plans of treatment in the United Kingdom and abroad. Dr. Macnamara, who replied, said the treatment of all cases of mental and nervous breakdown was undertaken in the three large base naval hospitals at Chatham, Haslar, and Plymouth. A medical officer who had made a specialty of such cases was detailed to each hospital, and he had as many beds allotted to him as were necessary. The treatment adopted was not to collect these cases into one ward or one building, but to separate them from each other as far as possible, treating them with other general cases of illness. So far this method of treatment had been followed by favourable results, but the number of cases of this type from the Fleet was not sufficiently large to enable more than a probable estimate of its value to be formed as yet. Later on it was hoped a detailed report of the results would be of value.

### Doctors' Chauffeurs.

Attention was directed by Mr. Rowlands to the inconvenience caused to medical men with a large rural practice by their chauffeurs being taken for military service; he asked the Under-Secretary for War to consider the advisability of these men being enrolled in the Royal Army Medical Corps and left in the employment of the doctors until absolutely required. Mr. Tennant, however, pointed out that there was urgency in the demand by the Army for motor-car drivers, but, subject to this, arrangements had been made for the consideration of individual cases.

### Time-Expired Men of the R.A.M.C.

A principle which has frequently been urged in the House of Commons is that young single men in the Royal Army Medical Corps fit for general service should be transferred to the infantry to make way for older men, who would rightly be given a preference as regards service in a Service less exposed to danger. Referring to this principle, Mr. Watt asked why in the interests of economy the training of time-expired men of the R.A.M.C. was not utilised, and Mr. Tennant replied that such transfers would not, of course, be made if they involved dislocation of the Service from which the transfer was made.

### Other Matters of Interest.

Smallpox.—Replying to Major Astor, the President of the Local Government Board stated that during the nine weeks ended May 27, 1916, seventy-nine cases of smallpox were notified in England and Wales. Sixty-two cases had been vaccinated and fifteen were unvaccinated; he had received no information as to the vaccinal condition of the other two cases. No person contracted smallpox subsequent to recent revaccination.

